# Advances in the Treatment of Acute Myeloid Leukemia: Implications for Low- and Middle-Income Countries

**DOI:** 10.3390/biomedicines13051221

**Published:** 2025-05-18

**Authors:** Michelle Morcos-Sandino, Sofia Isabel Quezada-Ramírez, Andrés Gómez-De León

**Affiliations:** Hematology Service, Facultad de Medicina y Hospital Universitario Dr. José Eleuterio González, Universidad Autónoma de Nuevo León (UANL), Av. Madero y Gonzalitos S/N, Mitras Centro, Monterrey ZC 64460, Nuevo León, Mexico; dra.michelle14@gmail.com (M.M.-S.); so.quez1507@gmail.com (S.I.Q.-R.)

**Keywords:** acute myeloid leukemia, newly diagnosed, relapsed or refractory, intensive chemotherapy, hypomethylating, BLC-2 Inhibitor, FLT3 inhibitors, IDH inhibitors

## Abstract

Acute myeloid leukemia (AML) presents a significant global health challenge due to its aggressive behavior and mortality rates. Traditionally, AML treatment has relied on intensive chemotherapy—anthracyclines and cytarabine. However, recent breakthroughs in targeted therapies are transforming clinical practices. This review examines current treatment strategies, including breakthrough therapies. Also, a global perspective on AML management includes the disparity in treatment availability, particularly the difficulties faced by low- and middle-income countries due to the high cost and restricted access to novel therapies.

## 1. Introduction

Acute myeloid leukemia (AML) poses a significant global health challenge due to its aggressive behavior and high mortality rates [1]. Advances in the understanding of its pathogenesis, characterized by complex genetic alterations affecting hematopoietic cell survival, proliferation, and differentiation involving transcription factors, kinase-driven signaling pathways, and epigenetic dysregulation [2], have led to major changes in its diagnosis and pharmacologic treatment. The global incidence of AML has increased over recent decades [1,3]. However, the management of AML in low/middle-income countries (LMICs) remains markedly different, primarily due to disparities in healthcare access, including the high cost and limited availability of treatments [4]. Addressing these barriers to timely diagnosis and effective therapy in resource-constrained settings is essential. This review will explore newly emerging diagnostic tools and therapeutic approaches, along with the challenges of their implementation in LMICs.

## 2. Diagnostic Work up and Risk Stratification

AML is a genetically heterogeneous disease. The foundation of its diagnosis lies in cytomorphological and cytogenetic analysis, necessitating molecular testing for accurate risk stratification. While conventional cytogenetics remains mandatory, about 40–60% cases lack cytogenetic abnormalities [5], resulting in variable clinical outcomes. In these cases, molecular profiling becomes essential, although not always feasible in resource-constrained settings.

### 2.1. Molecular Analysis

Several gene mutations must be assessed at diagnosis due to their critical prognostic and therapeutic implications. These include *FLT3-ITD*, *IDH1/IDH2*, *NPM1*, *CEBPA*, *TP53*, *CBFB::MYH11*, *RUNX1::RUNX1T1*, and *KMT2A* rearrangements [6,7]. Translocations can be revealed using fluorescence in situ hybridization (FISH) probes or RT-PCR assays and point mutations using multiple specific PCR assays; however, this strategy requires the performance of multiple separate tests. Next generation sequencing (NGS) is a massive parallel sequencing technique that analyzes multiple gene variants and is a valuable tool for risk assessment, therapy selection, and measurable residual disease (MRD) monitoring. The European LeukemiaNet (ELN) guideline for the diagnosis and management of AML, in its most recent revision, recommends the performance of NGS and the genetic assessment of at least 21 gene mutations including single gene mutations and structural variants [7,8]. The majority of clinical data used to validate the ELN-2022 risk classification are well-suited for young patients treated with cytarabine-based intensive chemotherapy, albeit not for HMA-treated patients [9]. ELN classification allows for the discrimination of patients with favorable risk, those who may be cured by chemotherapy alone, and adverse risk patients, those who are at considerable risk of adverse outcome regardless of current therapies and the availability of transplants. Intermediate risk patients have a better prognosis, and there is debate whether patient subgroups should be recommended to receive a first line allogeneic transplant recommendation [10]. Recently, an ELN classification for less intensively treated patients has been published where NPM1, IDH2, IDH1, and DDX41 mutations are considered favorable. The adverse risk category lists *TP53* mutations, present in 6–10% of de novo MDS/AML cases, as frequently observed in therapy-related disease (20–35% of cases) [11]. Mutant *TP53* is associated with poor over survival (OS) at a 2 year rate of less than 12.8% [12]. Therapy-related AML (t-AML) typically exhibits complex karyotypes and *TP53* mutations and is also linked with unfavorable prognosis [8].

#### 2.1.1. Molecular Testing Disparities in AML

LMICs have lower accessibility to diagnostic tests compared to high-income countries (HIC), which interferes with the best treatment of patients (see Table 1). Although NGS is more sensitive, it is still more costly when compared to older technologies [13,14]. Establishing and offering a sustainable NGS platform is challenging, requiring resources for its implementation including specialized training, significant financial investment, and infrastructure, sampling workflows, and quality monitoring [15,16]. Latin American patients have different molecular profiles [17,18] which impact their management. In a multicentric, retrospective analysis in Mexico, only 69.1% patients had karyotyping and less than 21% of cases were assessed for FLT3 or NPM1 mutations [19]. In a retrospective study from Brazil, 46% patients were unable to be classified due to absence of molecular testing [20]. An observational study conducted in Panama, Peru, and Colombia reported on 125 AML patients, of which 58% and 69% were not risk-stratified due to unavailability of cytogenetic and mutation assessments, respectively [21]. Colombia has previously reported similar rates, including only 62% of patients receiving molecular evaluations [22]. Finally, Chile reports 56.6% of patients had cytogenetic evaluations available [23]. In summary, around half of the patients in Latin America have access to cytogenetic or molecular evaluations, which hinders their adequate treatment. Reliance on karyotyping will continue until more sensitive testing becomes more cost-effective within the region.

A prospective study conducted in the United States (US) in the Connect MDS/AML registry reported that 98.8%, 95.4%, 75.9%, and 75.7% of patients were tested with flow cytometry, karyotyping, molecular testing, and FISH, respectively. Molecular testing was more commonly conducted in academic centers when compared to community centers (*p* < 0.001) [24]. A similar and more recent retrospective study reported that 95% of AML patients underwent cytogenetic testing and 91% received molecular testing. Molecular testing was more commonly conducted through NGS when compared to PCR [17]. Similarly, the Canadian Leukemia Study Group/*Groupe canadien d’étude sur la leucémie* (CLSG/GCEL) consensus recommends all newly diagnosed (ND)-AML adults undergo molecular assessment including FLT3 [25]. Spain has reported an incremental use of these tools for AML risk-stratification [26]. These reports confirm the integration of molecular testing into the routine management of AML patients in HIC.

#### 2.1.2. Testing Strategies for AML in Low-Resource Environments

In centers without access to comprehensive characterization, minimally required laboratory studies should include karyotype, FISH, or PCR for clinically actionable mutations, as well as those that determine or preclude the performance of allogeneic stem hematopoietic cell transplant (allo-SCT) in the presence of high-risk mutations. Therefore, in resource-constrained settings, karyotyping is an essential test in the evaluation of AML. Meanwhile, effective options for rapid screening of specific translocations associated with CBF-AML (Core Binding Factor), PML-RARA, and BCR/ABL include conventional cytogenetics, FISH, or RT-PCR [7]. Meanwhile, PCR may facilitate the identification of actionable mutations such as FLT3, NPM1, IDH1, and IDH2. The detection of NPM1 and TP53 mutations using immunohistochemistry (IHC) is under investigation and may serve as an alternative way to detect these mutations. While IHC is a widely available technique and may be beneficial for those where molecular testing is not feasible, this approach has not yet been validated [27,28,29].

**Table 1:** Key diagnostic challenges for AML in LMICs.

Diagnostic Modality	Common LMIC Limitation	Impact on Care/Recommendations
Morphology	Variable expertise, inconsistent quality control	Risk of misdiagnosis or delayed diagnosis. Inadequate assessment may hinder appropriate treatment selection. Standardization of morphology training and quality control is essential.
Flow Cytometry [30,31]	Limited access to lab centers, high implementation cost, need for trained personnel, difficulty with disease monitoring	Challenges in MRD assessment and risk stratification; limited ability to predict outcomes. If FCM is unavailable, immunophenotyping can be performed using IHC.
Cytogenetics	Unavailability, high cost, and limited lab infrastructure and expertise	Inability to perform standard risk stratification; delays when tests must be outsourced. IHC may be used to screen NPM1 and TP53 mutations, pending validation.
Molecular Testing [15,16]	Limited access to reagents, equipment, trained personnel, high cost	Inability to identify actionable mutations or perform risk stratification, treatment delays. If NGS is unavailable, use PCR or FISH to detect key mutations or translocations. IHC may be used to screen NPM1 and TP53, pending validation.

FCM = Flow Cytometry, IHC = immunohistochemistry, NGS = Next Generation Sequencing.

## 3. Frontline Treatment Strategies

### 3.1. Standard Induction Chemotherapy

For nearly five decades, the intensive chemotherapy (IC) regimen for remission induction has relied on the ‘7 + 3’ regimen which includes an anthracycline along with cytarabine (Ara-C). The most common anthracycline used is daunorubicin (DNR), administered at 60–90 mg/m^2^/day or idarubicin (IDA) 12 mg/m^2^/day for 3 days, while Ara-C (100–200 mg/m^2^/day) is typically administered in a continuous infusion for 7 days. This regimen induces a complete remission (CR) in approximately 70–80% of fit adults under 60 years, and 40–60% in those over 60 [32,33]. In a meta-analysis, IDA slightly improved CR and OS rates over DNR. Meanwhile, adverse events (AEs) remained comparable. However, both IDA and DNR have shown similar efficacy when DNR/IDA dose ratio is maintained at 5:1 ratio (e.g., 60 mg/m^2^, 12 mg/m^2^) [34].

### 3.2. Alternative Intensive Chemotherapy Regimens

Alternative induction regimens incorporating high dose cytarabine (HDAC) and anthracyclines—with or without nucleoside analogs—have been evaluated; however, none have consistently demonstrated superiority over the standard “7 + 3” regimen in unselected patient populations. Nonetheless, recent advances in subtype-specific therapies have yielded promising results. CPX-351, a liposomal formulation of DRN and cytarabine, significantly improved outcomes in secondary AML (sAML) compared to 7 + 3, and is now approved for frontline use in this setting [35]. Similarly, the addition of gemtuzumab ozogamicin (GO) to induction therapy has been shown to improve outcomes in CBF-AML [36,37,38]. While FLT3 inhibitors (FLT3i) such as midostaurin and quizartinib, when combined with 7 + 3, have demonstrated OS benefit in FLT3-mutated (FLT3m) AML [39,40] Venetoclax (Ven), when added to IC, they have been associated with enhanced response rates [41]. HDAC-based regimens have also shown superiority over standard dose Ara-C in younger patients, with improved CR (82.4% vs. 75.6%), 6-year OS (51.9% vs. 43.3%), and event free survival (EFS) (43.6% vs. 35.1%) rates, particularly in those with sAML [42].

The FLAG-Ida regimen (fludarabine, cytarabine, granulocyte colony-stimulating factor, idarubicin), as evaluated in the AML-15 trial, significantly reduced relapse rates (38% vs. 55%; *p* < 0.001) and improved relapse free survival (RLS) (45% vs. 34%; *p* = 0.01) compared to ADE (cytarabine, daunorubicin, etoposide), with a sustained survival benefit following allo-SCT, as reflected by 3-year OS of 58% vs. 37%, despite increased myelosuppression [43,44]. Cladribine-based regimens such as DAC and CLIA have demonstrated higher CR rates (67.5% vs. 56%) and MRD negativity (70%) in frontline settings [45,46,47,48]. The CLAG-M regimen achieved 71% CR with minimal early mortality [49]. Regarding GO, fractionated dosing (as in the ALFA-0701 trial) and single- or double-dose strategies (AML-18 trial) improved OS in patients without adverse risk features, though no OS difference was observed among transplant recipients [36,38]. Ongoing studies are evaluating novel combinations, including GO with midostaurin or FLAG-Ida, in both frontline and relapsed/refractory (R/R) disease [NCT04385290, NCT04050280]. Finally, long-term data on CPX-351 indicate a 5-year OS of 18% in older patients with sAML, compared to 8% with 7 + 3 [36]. A recent phase III trial in younger patients with high-risk AML or MDS reported superior RFS with CPX-351 versus FLAG-Ida (22.1 vs. 8.35 months) [50]. However, further studies are warranted to better define its role in AML harboring myelodysplasia-related mutations.

#### 3.2.1. Mutation-Targeted Intensification Strategies

##### FLT3-Mutated AML

FLT3 mutations are present in approximately 30% of AML cases, with internal tandem duplication (ITD) being the most common and associated with poor outcomes, while tyrosine kinase domain (TKD) mutations are less frequent and of uncertain prognostic value [51,52]. This has led to the development of FLT3i, classified by binding type: type 1 inhibitors (midostaurin, gilteritinib, crenolanib) target both active and inactive conformations, whereas type 2 inhibitors (quizartinib, sorafenib) bind the inactive form. Midostaurin, approved in 2017 based on the RATIFY trial, improved early outcomes but increased relapse rates after discontinuation; allo-SCT remained a key factor for long-term control [39]. Quizartinib, approved in 2023 (QuANTUM-First), showed sustained benefits in RFS and OS, particularly in patients who continued therapy without undergoing allo-SCT [40]. Sorafenib, studied in the SORAML trial, initially improved EFS and RFS, but post-relapse outcomes were worse, raising concerns about resistance following early exposure [53,54]. Gilteritinib, a second-generation type 1 FLT3i, is approved for R/R AML and has demonstrated high CR and MRD-negativity rates in frontline studies, with long-term survival benefits; phase III trial results in combination with chemotherapy are awaited [55,56]. Crenolanib, with limited c-KIT inhibition and a short half-life, showed favorable MRD and safety profiles in ND-AML patients, and is currently under evaluation in comparative trials against midostaurin in the frontline setting [57,58]. Currently, several front-line clinical trials are now evaluating other multi-targeted tyrosine kinase inhibitors, such as gilteritinib (NCT03836209) and crenolanib (NCT03258931), in comparison to midostaurin during induction and consolidation chemotherapy in untreated patients with mFLT3.AML.

##### Venetoclax Intensive Combinations

Early-phase studies combining IC with Ven report high response rates and deep remissions. The DAV regimen (7 + 3 + Ven) yielded a 91% CR rate and 97% MRD negativity, with manageable toxicity, though limited by small sample size and short follow-up [41]. FLAG-IDA-Ven in ND-AML showed ORR 99%, CRc 96%, and 89% MRD negativity, including in high-risk groups; however, TP53-mutated patients relapsed early [59]. CLIA-Ven achieved a 96% CRc rate in AML patients ≤ 65, with 90% MRD negativity, and 62% of responders underwent allo-SCT, with rates over 70% 2-year OS and DOR. Toxicity was manageable, with low early mortality [60,61]. These findings support CLIA-Ven as an effective induction option in fit, younger patients. Overall, these regimens demonstrate strong efficacy, supporting further evaluation in fit patients, though logistical challenges and risk of infection must be considered.

### 3.3. Are Intensive Chemotherapy Regimens Feasible Across All Resource Settings?

While IC regimens in AML have demonstrated high rates of CR and MRD negativity, suggesting enhanced anti-leukemic efficacy, their real-world feasibility in LMICs remains limited. Comparative studies between IC protocols are scarce, and while CR rates appear broadly similar, the resource burden associated with these regimens is substantial.

In HIC, the implementation of IC protocols is supported by robust healthcare infrastructure. For instance, in the United States, the average cost per induction episode ranges from USD 145,189 to USD 198,657, with consolidation therapy adding an additional USD 28,137 to USD 73,428 [62,63].These high costs are largely attributable to prolonged hospitalizations—necessary to manage post-chemotherapy complications such as infections, cytopenia, and organ dysfunction. A retrospective analysis of 642 AML patients undergoing IC in the U.S. revealed that all patients required at least one hospital admission, with a median of two stays averaging 16 days each. Notably, 64.3% were treated in large tertiary centers (≥500 beds). The median inpatient hospital cost was $83,440 per patient, with ICU-related expenses reaching a median of $16,550 [64].

In contrast, LMICs often lack the infrastructure to safely administer these regimens. Barriers include limited access to supportive care (e.g., antimicrobial prophylaxis, transfusion support, ICU beds), inadequate insurance coverage, and a shortage of inpatient capacity. In many centers, hospital bed availability is constrained, and prolonged admissions for chemotherapy recovery are not feasible [4,65,66]. Furthermore, a high proportion of AML patients in LMICs are uninsured, making the economic burden of IC prohibitive. This disparity is further exemplified by an Indian cohort in which unfit patients received IC due to the unavailability of alternative treatments. These patients experienced higher induction-related mortality and reduced OS, underscoring the potential harm of applying intensive protocols outside appropriate settings [67].

Thus, while IC remains a cornerstone of AML treatment in well-resourced settings, its widespread use in LMICs is often neither practical nor safe. Tailoring treatment strategies to local healthcare capacities and expanding access to lower-intensity regimens or novel oral agents are critical steps toward equitable AML care.

## 4. Post-Remission Consolidation Therapy

The primary goal of post-remission therapy is relapse prevention. Key factors in decision-making include donor availability, patient fitness, ELN risk (intermediate to adverse), and MRD clearance post-induction. Allo-SCT is the most effective consolidation, particularly for patients with adverse-risk mutations or cytogenetic abnormalities, and is recommended for those achieving first CR. For patients with favorable to intermediate-risk profiles, or when allo-SCT is not feasible, consolidation with intermediate-dose cytarabine (IDAC, 1–1.5 g/m^2^) or HDAC (≥2 g/m^2^) remains standard [7]. While the optimal regimen is still debated, IDAC seems less toxic without compromising OS or DFS. HDAC is traditionally given on days 1, 3, and 5 (HDAC-135) for a total number of 4 cycles. However, the condensed HDAC-123 regimen (days 1–3) is preferred due to faster hematologic recovery, fewer infections, shorter hospital stays, and potential for outpatient administration, optimizing healthcare resources and thus is our preferred approach [68,69].

### 4.1. Allogeneic Stem Cell Transplantation

Allo-SCT is the only curative approach for many AML patients, primarily due to the graft-versus-leukemia effect [70], and is used particularly for those with adverse-risk features, relapsed disease, or persistent MRD. In intermediate-risk AML, it is advised when a matched donor is available [7,71]. The choice of donor, graft source, and conditioning intensity depends on patient condition and institutional resources. Advances such as MRD-guided timing, reduced-intensity conditioning (RIC), and wider use of haploidentical donors have expanded access while improving outcomes [72,73]. In MRD-positive patients, myeloablative conditioning (MAC) offers superior disease control, whereas RIC is adequate for MRD-negative individuals, reducing toxicity [74,75,76]. Matched sibling donors (MSD) remain the preferred source, though matched unrelated donors (MUD) yield similar outcomes [77]. Refinements in transplant protocols have also improved haploidentical SCT, with emerging data supporting similar survival rates [78,79,80]. The success of post-transplant cyclophosphamide has enabled broader use of haploidentical SCT, with emerging evidence supporting its long-term efficacy despite initial concerns over rejection and severe graft-versus-host disease (GVHD) [81,82].

### 4.2. Allogeneic Stem Cell Transplant in LMICs

#### 4.2.1. Health System and Logistical Barriers to Access Allo-SCT

Although allo-SCT is an old, potentially curative treatment for AML, its use is unevenly distributed worldwide [72,83]. In 2016, allo-SCTs were performed worldwide, with the highest volumes reported in Europe (6238), the South-East Asia/Western Pacific region (4572), and North America (3223). In contrast, significantly fewer procedures were conducted in South America (502) and the African/Eastern Mediterranean region (430). Utilization rates among patients with AML were highest in Europe and North America, reaching approximately 18%, while much lower rates were observed in South America (4.2%) and Africa/Eastern Mediterranean (2.7%) [84]. These disparities are driven by differences in healthcare infrastructure, national income, donor registries, and trained personnel [85,86,87]. Greater distance from transplant centers is also linked to higher 1-year non relapse mortality (NRM) [88]. LMICs face greater barriers due to fewer transplant centers [83,85,89], often located in the private sector [90]. Furthermore, a lack of formal training programs, particularly in Latin America, exacerbates workforce gaps [91]. Resource limitations also affect post-transplant care such as limited caregiver support, especially for adults needing rotating care [92,93,94]. Inconsistent availability of antimicrobials, blood products, chemotherapy, and ICU support all compromise outcomes, especially in centers with less experience [95,96]. This is reflected in varying OS and early mortality after allo-SCT in relation to facility type and experience [97]. Moreover, unavailability of key drugs influence patient outcomes, such as delayed antibiotic administration, which correlates with higher rates of mortality [98,99].

To reduce costs and improve access, cost-saving strategies have been adopted in some LMICs, including outpatient transplants [89,90,91,100,101,102], use of biosimilar drugs, haploidentical donor programs, avoidance of stem cell cryopreservation [85], and use of leukoreduced blood [92,103]. In our institution, we are exploring the use of reduced intensity conditioning regimens in patients with AML and MRD negative disease undergoing allo-HCT in an effort to reduce complications while preserving the graft-versus-leukemia effect [104], based on retrospective international data that has shown comparable outcomes for MAC vs. RIC in MRDnegative patients [74,75]. These measures aim to expand access while adapting to local resource constraints.

#### 4.2.2. Disparities in Donor Availability for Allogeneic Transplantation

In HIC, HLA typing at diagnosis is standard practice; however, fewer than 30% of AML patients have a MSD, necessitating alternatives such as haploidentical or MUD. Disparities exist between Latin America, Europe, and North America, and even within each country’s registry [105,106]. In a US registry, 75% of white patients of European descent in U.S. registries find a match, compared to only 16% of Black Latin American patients [107]. Early donor identification improves outcomes: a U.S/Canada study using buccal swabs for upfront HLA typing in high-risk AML increased allo-SCT in first remission from 40% to 65%, with improved 2-year OS and reduced time to transplant [108]. In contrast, countries face barriers to timely allogeneic transplantation due to limited infrastructure, funding, and delayed referrals. In Mexico, only one-third of eligible patients undergo timely HLA testing due to relapse, financial barriers, limited access, and donor scarcity [109,110]. Voluntary HLA typing is increasing globally but remains limited in LMICs [99]. MUD use remains low in Latin America (25%) compared to Europe (56%) [105], with similar trends in Brazil, Chile, and Colombia [111]. Given these constraints, haploidentical donors have emerged as a more accessible and cost-effective option in many settings, and this is now the most common form of allo-HCT performed in the region [112].

#### 4.2.3. Adapting Stem Cell Transplantation to Low-Resource Settings

Expanding access to stem cell transplantation in AML requires early HLA typing, broader donor registries, and efforts to reduce racial disparities in donor matching. Although national registries exist in Latin America, like REDOME in Brazil, INCUCAI in Argentina, DONORMO and NMDP in México, and DKMS in Chile, access remains limited overall [112]. Equitable resource distribution and education are critical for improving outcomes.

In low-resource settings, cost-saving strategies include outpatient transplants [100,101,102], use of biosimilars, haploidentical donors, avoidance of stem cell cryopreservation [85], and leukoreduced blood products [103]. RIC regimens provide safer alternatives when intensive care is limited [102,104,113,114]. Telemedicine supports remote monitoring and donor coordination [115]. However, a persistent barrier is the shortage of trained transplant professionals, highlighting the urgent need to invest in workforce development in LMICs [91].

## 5. Patients Ineligible for Intensive Treatment

Treating older adults is more challenging than treating younger patients, primarily due to higher risk disease, comorbidities, and decreased physiological reserve, which often limit tolerance to intensive chemotherapy. Before the advent of Ven, hypomethylating agents (HMAs) like Azacitidine (Aza) or Decitabine (DEC) were the standard of care for this population. These agents demonstrated improved OS compared to supportive care or low dose cytarabine (LDAC), and offered survival outcomes comparable to IC in select older patient, despite lower CR rates and with more favorable safety profiles [116,117].

### 5.1. Targeted Therapies and Mutation-Driven Approaches

The identification of actionable mutations such as *IDH1*, *IDH2*, *KMT2A*, and *FLT3* in AML has enabled the development of mutation-specific therapies. However, the optimal therapeutic strategy for these molecular subgroups remains under investigation. Current approaches include HMA combined with targeted inhibitors, HMA with Ven, or triple regimens incorporating both. The role of targeted monotherapy is also being explored, with ongoing trials evaluating efficacy, sequencing, and long-term outcomes.

#### 5.1.1. Venetoclax-Based Treatments

The combination of HMAs with Ven became the standard of care for unfit patients following the VIALE-A and VIALE-C trials, which demonstrated improved remission rates and OS compared to HMA alone [118,119]. Long-term follow-up of VIALE-A confirms durable benefit with Aza-Ven [119].

Ven sensitivity is associated with *NPM1*, *IDH1/2*, and *RUNX1* mutations, while resistance has been linked to *FLT3-ITD*, *TP53*, and RAS pathway mutations [120]. The ELN genetic risk model has limited predictive value in older patients, prompting the development of the molecular Prognostic Risk Signature (mPRS), recently validated for Ven-based regimens [10,121]. In parallel, the MACS-score (Mediators of Apoptosis Combinatorial Score)—a flow cytometry-based biomarker that assesses BCL-2, MCL-1, and BCL-xL protein expression in leukemic stem cells—has shown a >97% predictive value for EFS [122]. Although external validation is pending, it offers a potential alternative in resource-limited settings without access to molecular diagnostics and is currently under evaluation at our center.

Effective risk stratification remains critical for optimizing Ven-based therapy, especially in high-risk subsets such as m*TP53*-AML, where HMA-Ven may confer limited benefit despite increased cost and toxicity.

##### Venetoclax Dosing

Ven combined with HMAs is effective in AML but frequently leads to myelosuppression and infectious complications. The standard regimen includes Ven 400 mg daily for 28 days with Aza (75 mg/m^2^ for 7 days) or DEC (20 mg/m^2^ for 5 days). However, the VIALE-A trial permitted Ven reduction to 21 days during cycle 1 to mitigate cytopenias without compromising OS [123].

In clinical practice, dose modifications and delays are common. Retrospective analyses suggest that shortened Ven schedules (7–14 days) may preserve efficacy while reducing toxicity. A study of untreated AML patients receiving Aza or LDCA plus Ven across 14-, 21-, and 28-day schedules found no significant differences in remission rates, MRD negativity, or outcomes in high-risk subgroups, though the 28-day regimen incurred twice the drug cost [124].

An alternative regimen of Ven 100 mg daily for 14 days with Aza achieved favorable responses and lower infection rates (17%) compared to the 64% infection rate in VIALE-A [125]. In patients over 80 years, shorter Ven exposure (≤14 days) was associated with better overall survival (24 vs. 8 months) and comparable response rates to longer schedules. The study proposed Ven 400 mg for 14 days or 200 mg for 21 days on a 35-day cycle as an effective and safer alternative. The study suggested VEN 400 mg for 14 days or 200 mg for 21 days, every 35 days, as optimal regimen [126]. These findings underscore the importance of tailoring Ven duration to optimize the balance between efficacy and tolerability, particularly in older or frail patients.

#### 5.1.2. Emerging Venetoclax Combinations Under Investigation

Recent treatment strategies incorporate Ven, alongside standard chemotherapy, reformulated combinations, and less intensive regimens, to improve outcomes and reduce toxicity. Several ongoing phase II–III trials exemplify this shift. The MyeloMATCH trial (NCT06917911) evaluates early Ven or GO with DRN and Ara-C in core binding factor AML. NCT06903702 compares Aza plus Ven to standard induction in ND-AML, focusing on 1-year RFS. NCT06770257 studies a liposomal DRN/Ara-C formulation combined with Ven for improved synergy and tolerability. In maintenance, NCT06765928, assesses selinexor with Ven in AML and MDS. For R/R disease, NCT06763666 compares CLAG-VEN to standard CLAG. In older adults, NCT06744556 tests hydrocortisone, Ara-C, and Ven (HAV) regimen versus anthracycline protocols. NCT06050941 evaluates a reduced-intensity “3 + 5” IDA/Ara-C regimen. Lastly, NCT05356169 examines Ven with or without IC in a large, randomized cohort. Collectively, these studies highlight the shift toward personalized, risk-adapted AML therapy.

#### 5.1.3. AML with Mutant IDH1/2

**Figure 1:**
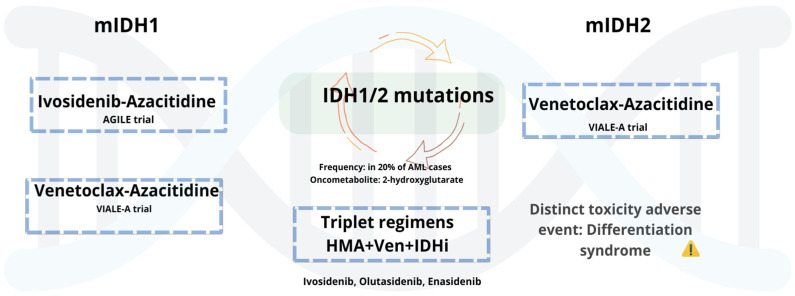
mIDH1/2 AML: Low-intensity regimens and emerging triplets.

Mutations in *IDH1* and *IDH2* occur in approximately 6–10% and 12–15% of AML cases, respectively [127], and have led to the development of targeted therapies with selective inhibitors (see Figure 1). Ivosidenib (Ivo), an oral mIDH1 inhibitor, received approval in untreated AML patients based on the AGILE phase 3 trial, where Ivo plus with Aza (Ivo + Aza) significantly improved outcomes compared to Aza alone [128]. Updated follow-up confirms sustained benefit, with a median OS of 29.3 months. Benefits included improved transfusion independence and hematologic recovery, with comparable toxicity to monotherapy [129]. Olutasidenib (Olu), an oral mIDH1 inhibitor, has shown durable responses in R/R AML. In a 5-year follow-up, 35% achieved CR with a median DOR of 25.3 months. Median OS was 11.6 months, increasing to 16.2 months in patients previously treated with Ven. Over 40% of transfusion-dependent patients achieved independence. Common AEs included differentiation syndrome, febrile neutropenia, and gastrointestinal toxicity [130]. These results support ongoing investigation of Olu in both frontline and salvage settings. Enasidenib (Ena), a selective IDH2 inhibitor, is approved for R/R mIDH2 AML. In the IDHENTIFY trial, Ena showed no survival advantage over standard care, though over 31% of transfusion-dependent patients achieved independence [131]. While in the frontline setting, the AG221-AML-005 trial evaluated Ena + Aza in ND, unfit patients. Ena plus Aza improved response rates (ORR 74%, CR 54%) compared to Aza alone, but without a significant OS benefit. Increased gastrointestinal and hematologic toxicity and differentiation syndrome were observed [132]. Collectively, these agents demonstrate clinical activity in *IDH*-mutated AML, with notable benefits in response and transfusion independence. However, limitations in survival impact, particularly with Ena, underscore the need for further combination strategies and biomarker-driven patient selection.

#### 5.1.4. Combinations and Emerging Therapeutic Pathways of Mutated IDH-AML

Combining targeted agents offers a strategy to overcome resistance in AML. A phase Ib/II trial (NCT03471260) assessed Ivo and Ven, with or without Aza, in mIDH1 AML. The triplet (Ivo + Ven + Aza) achieved a CRc of 90%, versus 83% with Ivo + Ven. One-year survival was higher with the triplet (90% vs. 50%), especially in ND or MDS/MPN patients. Severe AEs, mainly infections, were similar across groups [133]. Another ongoing phase Ib/II trial (NCT04774393) is evaluating decitabine/cedazuridine (ASTX727) and Ven with either Ivo or Ena in mIDH1/2 AML. Preliminary pooled data are encouraging, pending full results. The I-DATA phase II trial (NCT05401097) is testing sequential regimens, comparing Ven + Aza followed by an IDH inhibitor plus Aza, versus the reverse. A phase I/II trial (NCT03515512) assessed Ena as maintenance therapy after allo-SCT in IDH2-AML. Preliminary relapse activity prevention was reported. While definitive data on adding IDH inhibitors to IC are still pending, early-phase results suggest that combining Ivo or Ena with IC is feasible and well tolerated in ND mIDH1/2 AML [134]. Ongoing trials are testing these combinations in both frontline and R/R settings (NCT03839771, NCT02632708, NCT04493164). In R/R AML, additional strategies are being explored. Ena is under investigation with the MEK inhibitor cobimetinib to counter RAS-driven resistance, often present in IDH2-mutated cases (NCT05441514). A phase II study is evaluating olaparib, a PARP inhibitor, in mIDH1/2 AML. Meanwhile, HMPL-306, a dual IDH1/2 inhibitor, is being compared to salvage IC (NCT06387069). These trials aim to refine the role of IDHi in high-risk and refractory populations, potentially offering alternatives for patients with co-mutations or limited options.

### 5.2. FLT Mutated AML and FLIT Inhibitors: Low Intensity and Maintenance Therapies

FLT3 inhibitors (FLT3i) have been evaluated in low-intensity regimens for unfit AML patients with m*FLT3*, though survival benefits remain unproven, and no targeted therapy is currently approved in this setting. In a phase 3 trial, Aza plus Gilteritinib (Gilt) improved composite complete remission (CRc) rates compared to Aza alone (58.1% vs. 26.5%) but failed to meet OS and EFS endpoints. AEs, particularly gastrointestinal and cardiac toxicities, were more frequent with this combination [135].

Preclinical data suggest synergy between FLT3i and BCL-2 inhibitors [136]. In the R/R setting, the Ven-Gil achieved a 75% CRc rate, including FLT3-ITD burden reduction in 60% of patients. Outcomes were favorable even among those with prior FLT3i exposure, with 60% of transplanted patients alive at 17.5 months. However, nearly half required dose interruptions due to toxicity, highlighting the need for improved management strategies [137]. Quizartinib has also shown activity in this population. In a randomized study, quizartinib plus LDCA improved CRc (38%) and OS (17.5 vs. 5.1 months) compared to LDAC alone, with acceptable cardiac safety despite increased grade 3/4 events [138].

FLT3 inhibitors have also demonstrated efficacy as post-transplant maintenance. In the MORPHO trial, gilteritinib reduced relapse risk in patients with measurable residual disease pre- or post-HCT (HR 0.515; *p* = 0.0065), with over half completing two years of therapy despite frequent cytopenias and infections [139]. Similarly, the SORMAIN trial showed that sorafenib reduced relapse (CIR 15% vs. 36.3%) and improved OS (72% vs. 55.9%) post-transplant. However, tolerability issues and its off-label use limit widespread adoption [140].

#### Combinations and Emerging Therapeutic Pathways of mFLT3-AML

**Figure 2:**
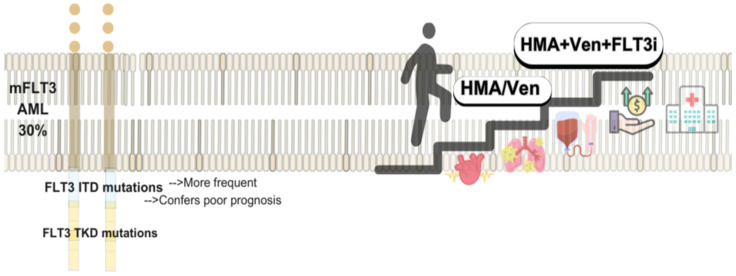
Targeting FLT3-AML: Evolving Low-Intensity Regimens.

Triplet regimens combining FLT3i have shown improved survival and response rates in frontline treatment, leading to prospective studies assessing combination strategies (see Figure 2). Although, optimal dosing, scheduling, and treatment sequencing remain uncertain, particularly in reducing prolonged myelosuppression. In phase 1b/2 study (NCT04140487), Aza, Ven, and Gil achieved a CRc of 96% and MRD negativity in 93% of ND-AML patients. FLT3-ITD clearance was seen at 65% after four cycles. With 19.3 months of follow-up, 18-month RFS and OS were 71% and 72%, respectively [137]. A separate phase 1/2 trial evaluated DEC, Ven, and Quizartinib in both R/R and ND patients. Overall CRc was 60%, but higher in FLT3i-naïve patients (71%). Median OS was 6.3 months overall and 10.3 months in the FLT3i-naïve group. In the untreated cohort (median age 75), CRc reached 95%, with 1-year OS of 78%. The RP2D for Quizartinib was established at 30 mg/day. Median hematologic recovery was 40 days; day-60 mortality was 13% [141]. Ongoing trials (e.g., NCT03661307, NCT05520567) are further evaluating these triplet strategies to optimize dosing and validate long-term outcomes.

## 6. Biology-Driven Therapeutic Advances: CAR-T Cell Therapy, Menin Inhibitors, and Targeted Strategies for High-Risk Population

Novel therapies are crucial as many patients’ diseases develop resistance to conventional therapy. Menin inhibitors target the protein product of the gene *MEN1*, a chromatin adaptor which interacts with the methyltransferase *MLL1* amongst other proteins. In many types of malignancies, it has been found that *MEN1* mutations play key roles in tumor development through both transcriptional activation and repression. However, in AML it seems that menin’s role is mainly as a cofactor for other fusion proteins, usually involving *MLL1*. It is through this interaction that expression of *HOX* genes (i.e., *HOX7*, *HOX8*) is increased, contributing to leukemogenesis. Many AML subtypes —such as AML with MLL1 rearrangements and mutated NPM1—may benefit from menin inhibitors, as HOX gene activation is a key driver [142]. To date, only one menin inhibitor has received approval by the FDA for R/R AML treatment: Revumenib in 2024 [143]. Revumenib inhibits the interaction between menin and lysine methyltransferase 2A (KMT2A). Phase 2 demonstrated an OR of 63%. However, grade ≥ 3 AEs occurred, such as febrile neutropenia, differentiation syndrome, and QT segment prolongation [144]. Other menin inhibitors are in development, like Ziftomenib, which has successfully undergone phase 1 studies and will soon move on to phase 2 trials [145]. Trials evaluating the combination of menin inhibitor with chemotherapy, HMAs, or Ven are ongoing (NCT05761171, NCT05326516, NCT05886049, NCT05360160, NCT06284486).

Chimeric antigen receptor T (CAR-T) cell therapy has grown momentum as a potential therapy line due to the outcomes seen in other hematological malignancies, especially acute lymphoblastic leukemia. However, its utility for AML treatment is yet to be demonstrated. Contrary to ALL, AML does not possess a surface marker specific to the malignant cells, which complicates the design of an efficient CAR target. Furthermore, as they are investigational and costly products, their translation into LMICs everyday clinical practice will probably lag. Nonetheless, current clinical trials are evaluating how this new tool fits into the current SOC for AML. Some investigational targets are as follows: CD123, CD33, CD19, CLL1 (CD371), and IL-1RAP, amongst others [146,147].

TP53 mutations are among the most adverse prognostic markers in AML, primarily due to inherent resistance to chemotherapy and limited efficacy of current treatment strategies. In the VIALE-A trial, patients harboring TP53 mutations derived no meaningful benefit from Aza + Ven, raising concerns about the added toxicity and cost without survival gain [118]. Similarly, responses to Ven combined with intensive regimens such as CLIA or DAV have been limited in this subgroup [41,61]. Although a small series reported MRD negativity in TP53-mutated patients treated with FLAG-Ida plus Ven, long-term outcomes remained poor [59]. The phase III trial comparing CPX-351 to conventional 7 + 3 in high-risk AML also demonstrated that patients with TP53 mutations had dismal outcomes irrespective of the regimen used [148]. Furthermore, the benefit of allo-SCT in this setting remains uncertain, as relapse and OS continue to be suboptimal even after transplant. Novel therapies targeting non-apoptotic mechanisms are being explored. Magrolimab, an anti-CD47 monoclonal antibody that enhances macrophage-mediated phagocytosis, has shown early promise. In a phase Ib study combining magrolimab with Aza in previously untreated high-risk MDS, 40% of TP53-mutated patients achieved CR, with a median OS of 16.3 months [149]. The ENHANCE phase III trial (NCT04313881) is ongoing to evaluate this combination further. However, in the ENHANCE-3 phase III trial in ND, unfit AML, the addition of magrolimab to Aza + Ven failed to improve survival or response rates and was associated with an increased incidence of fatal AEs, leading to early study termination [149]. Overall, TP53-mutated AML remains a major therapeutic challenge, underscoring the need for novel, biology-driven approaches that can overcome its intrinsic resistance. Glasdegib is a Hedgehog pathway inhibitor approved for use with LDC in unfit patients. It has shown particular benefit in those with secondary AML. In the BRIGHT AML 1003 trial, glasdegib plus LDCA nearly doubled the median OS compared to LDC alone [150]. Its favorable safety profile supports its role as a low-intensity treatment alternative.

## 7. Alternatives in Constrained Settings for AML Patients with Targetable Mutations

In resource-limited settings, treatment of AML with targetable mutations requires an individualized approach based on drug availability, patient-specific factors, and access to clinical trials. When IDH or FLT3 inhibitors are not accessible, Ven-based regimens offer effective alternatives. When mIDH1s, such as Ivo or Olu, are unavailable, Ven-based regimens offer effective alternatives. Subgroup data from the VIALE-A trial show that Aza + Ven significantly improves outcomes in patients with mIDH1/2 mutations. For mIDH1 AML, median OS was 10.2 vs. 2.2 months, and CRc was 56.5% vs. 9.1% compared to Aza alone. For mIDH2 AML, median OS reached 27.5 vs. 13 months [151]. When azacitidine is not accessible, LDAC plus Ven may serve as a viable option. In the VIALE-C trial, patients with IDH1/2 had a median OS of 11.2 months, though limited by small sample size [119]. Similarly, for unfit patients with mFLT3-AML, Ven plus Aza has demonstrated efficacy when FLT3 inhibitors or triplet combinations are not feasible. In VIALE-A, CRc rates were 63% for mFLT3-ITD and 77% for FLT3-TKD, with median OS of 9.9 and 19.2 months, respectively [151]. These findings support the use of Aza/Ven as a practical and effective backbone in constrained settings for patients with IDH or FLT3 mutations when targeted agents are unavailable. Moreover, tailoring Ven regimens—by adjusting dose or treatment duration—may reduce toxicity, improve tolerability and quality of life, and lower costs. Dose modifications can be guided by clinical context, including mutation profile, cytogenetics, prior MDS, response depth, MRD status, and overall patient fitness. While these alternative strategies show promise, further research is needed to optimize treatment in constrained environments and support evidence-based decisions in the absence of targeted therapies.

### Access to Clinical Trials in LMICs

Cancer treatment poses a financial burden for patients with hematological malignancies [62]. Clinical trials provide access to novel treatments that might otherwise be unavailable. An analysis of 3345 leukemia trials found only 4.8% included sites in LMICs. Late-phase trials were more common in these regions (Phase III: 36.2% vs. 9.0%; Phase IV: 6.3% vs. 2.2%), while early-phase studies were underrepresented (Phase I: 6.3% vs. 27.6%; Phase II: 45.6% vs. 55.0%) compared to HICs [152]. Since late-stage trials often test already-approved treatments, patients in LMICs experience delays in accessing new therapies. Increasing early-phase trials in these regions is key to expanding access, improving outcomes, and ensuring globally applicable research. On the other hand, a study of patients with hematological malignancies, found no significant differences in financial burden between patients treated in clinical trials and those receiving standard of care [72]. This highlights the economic challenges faced by all patients. Finally, cultural barriers, poor understanding of the clinical trials, and potential lack of economical compensation are patient-related barriers faced in resource-constrained settings [73].

## 8. Strategies to Reduce Costs in AML: Our Approach

**Figure 3:**
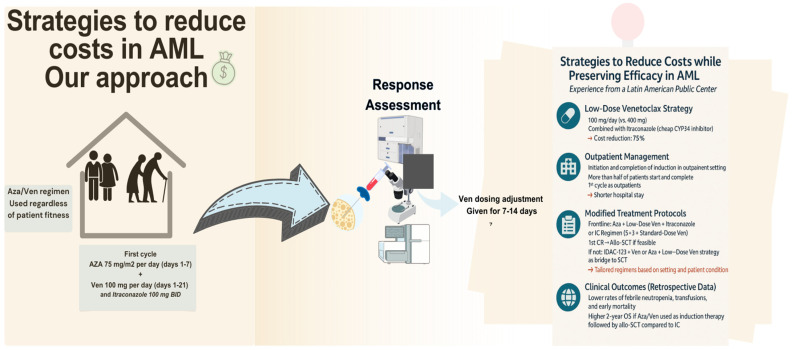
Cost-reduction strategies for AML management in a LMIC center.

Our center is a public tertiary facility in Latin America. Our population has a scarcity in oncologic treatment, leaving the elderly, particularly those with acute leukemias, most affected. Only about 2.8% of the population has access to private healthcare which offers coverage for new therapies. Meanwhile, 26.5% of the population lacked any form of insurance. Consequently, access to new treatments is largely limited to those with private insurance or the financial means to pay out-of-pocket [153]. Figure 3 summarizes our strategies for cost reduction in AML patients.

In our practice, considering the OS benefit in unfit patients demonstrated in the VIALE-A trial, for the frontline setting, we favor the combination of Ven and Aza for all patients, regardless of fitness. Furthermore, our approach uses a lower dose of Ven (100 mg) than the standard (400 mg). The rationale for giving a lower dose is that Ven is predominantly metabolized by CYP3A [106]. The pharmacological interaction between Ven and Posaconazole (a strong cytochrome P450 3A (CYP3A) inhibitor) resulted in a 93% increase in Cmax (maximum serum concentration) and a 155% increase in AUC (area under the curve) for a 100 mg dose of Ven when combined with posaconazole [154]. This interference within the pharmacokinetic interaction increases Ven plasma concentration by reducing its metabolism, allowing us to lower both dosage of Ven and the treatment costs by up to 75%. However, our strategy uses itraconazole, which is more affordable in our country than posaconazole or voriconazole [155].

Based on the aforementioned, this strategy is being used in our center (NCT05048615) [156]. Preliminary results in ND AML patients reported the feasibility and safety of low-dose Ven plus itraconazole with Aza. Notably, this has allowed us to start and complete the first induction in an outpatient setting (60 and 77.7%, respectively)., with short hospitalization stays (median 7 days) and without compromising efficacy (response rates 1st cycle: CRc 53.9%, 2nd cycle: 85.7%) and safety [109] compared to those reported in landmark studies [118,157]. Our regimen includes Ven at a dose of 100 mg once daily for 21 days, administered alongside itraconazole 100 mg twice daily and a fixed dose of Aza 100 mg once daily for 7 days. In subsequent cycles, we have reduced the frequency of Ven administration to 7–14 days for patients in remission.

On the other hand, when an IC induction regimen is preferred over Ven/Aza, it is based on a “5 + 3” combined with Ven regimen: IDAC, anthracycline (primarily mitoxantrone), and Ven 400 mg daily for 7 days (we use a standard Ven dose to avoid drug-to-drug interactions with IC). Upon first CR1, we offer allo-SCT if feasible; alternatively, we may provide chemotherapy using a condensed IDAC-123 regimen (1.5 g/m^2^ twice daily) alongside Ven (400 mg daily for 7 days) or low dose Ven + Aza regimen.

Although a formal healthcare resource expenditure or budget impact analysis has not been performed, these approaches may significantly reduce costs through outpatient induction, low-dose Ven along with azole use, lower hospitalization rates and stay length, and transfusion needs, especially when using Ven (100 mg) paired with itraconazole, contributing to Ven cost savings at a minimum of up to 75% [156,158]. For instance, we retrospectively analyzed 83 patients at our center (2016–2023) who underwent induction with either low-dose Ven/Aza or IC. Those who received induction with Ven/Aza had lower rates of febrile neutropenia, transfusions, and hospitalization, with early mortality at 30 and 60 days (2.4- and 3-fold reduction) compared to IC. Notably, patients that underwent allo-SCT and received Aza/Ven as induction had higher 2-year OS rates, 80% vs. 35.5%, than those who underwent IC followed by allo-SCT [158]. Additionally, an observational study evaluated adults with AML treated with Ven-based regimens, specifically in Latin-American public and private health centers (Mexico and Peru). Interestingly, Ven/Aza was the preferred regimen (60%) combined with azoles (83%), and the median maximum dose of Ven was 200 mg (range 100–600 mg) [159]. Although there is a lack of randomized studies, both of these strategies showed that Ven dose reduction and a low-priced azole, such as itraconazole, might be a reasonable approach in resource constrained settings.

## 9. Conclusions

In this review, we aimed to provide a general treatment perspective for different subgroups of patients in both frontline and relapsed/refractory settings, considering their fitness to receive intensive chemotherapy, low-intense treatment, and targeted therapies for specific mutations. Unfortunately, novel therapies are not universally available, and treatment approaches are becoming more personalized. However, certain strategies might be employed to enhance AML care even in resource-constrained settings.
